# Decentralised clinical trials in multiple sclerosis research

**DOI:** 10.1177/13524585221100401

**Published:** 2022-06-23

**Authors:** Afagh Garjani, Brandon Jun-Yu Liu, Christopher Martin Allen, Douglas David Gunzler, Stephen William Gerry, Sarah Marie Planchon, Roshan das Nair, Jeremy Chataway, Emma C Tallantyre, Daniel Ontaneda, Nikos Evangelou

**Affiliations:** Mental Health and Clinical Neurosciences Academic Unit, School of Medicine, University of Nottingham, Nottingham, UK/Academic Neurology, Nottingham University Hospitals NHS Trust, Nottingham, UK; School of Psychology, University of Nottingham, Nottingham, UK; Mental Health and Clinical Neurosciences Academic Unit, School of Medicine, University of Nottingham, Nottingham, UK/Academic Neurology, Nottingham University Hospitals NHS Trust, Nottingham, UK; The MetroHealth System and Case Western Reserve University, Cleveland, OH, USA; Centre for Statistics in Medicine, Nuffield Department of Orthopaedics, Rheumatology and Musculoskeletal Sciences, University of Oxford, Oxford, UK; Mellen Center for Multiple Sclerosis, Cleveland Clinic, Cleveland, OH, USA; Mental Health and Clinical Neurosciences Academic Unit, School of Medicine, University of Nottingham, Nottingham, UK/Institute of Mental Health, Nottinghamshire Healthcare NHS Foundation Trust, Nottingham, UK; Queen Square Multiple Sclerosis Centre, Department of Neuroinflammation, UCL Queen Square Institute of Neurology, Faculty of Brain Sciences, University College London, London, UK/National Institute for Health Research, University College London Hospitals Biomedical Research Centre, London, UK/MRC CTU at UCL, Institute of Clinical Trials and Methodology, University College London, London, UK; Helen Durham Neuro-Inflammatory Unit, University Hospital of Wales, Cardiff, UK/Division of Psychological Medicine and Clinical Neurosciences, Cardiff University, Cardiff, UK; Mellen Center for Multiple Sclerosis, Cleveland Clinic, Cleveland, OH, USA; Mental Health and Clinical Neurosciences Academic Unit, School of Medicine, University of Nottingham, Nottingham, UK/Academic Neurology, Nottingham University Hospitals NHS Trust, Nottingham, UK

**Keywords:** Decentralised, clinical trial, randomised controlled trial, multiple sclerosis, remote, digital

## Abstract

Randomised controlled trials (RCTs) play an important role in multiple sclerosis (MS) research, ensuring that new interventions are safe and efficacious before their introduction into clinical practice. Trials have been evolving to improve the robustness of their designs and the efficiency of their conduct. Advances in digital and mobile technologies in recent years have facilitated this process and the first RCTs with decentralised elements became possible. Decentralised clinical trials (DCTs) are conducted remotely, enabling participation of a more heterogeneous population who can participate in research activities from different locations and at their convenience. DCTs also rely on digital and mobile technologies which allows for more flexible and frequent assessments. While hospitals quickly adapted to e-health and telehealth assessments during the COVID-19 pandemic, the conduct of conventional RCTs was profoundly disrupted. In this paper, we review the existing evidence and gaps in knowledge in the design and conduct of DCTs in MS.

## Introduction

Randomised controlled trials (RCTs) are an essential component of modern healthcare, ensuring that new interventions are safe and efficacious before their introduction into clinical practice. RCTs, however, are expensive, time-consuming and burdensome to participants, investigators and funders, highlighting a need for innovations that reduce their high ‘failure’ rate.^1–4^ Success may be threatened, for example, by lack of funding due to prohibitively high costs,^1,3^ low statistical power due to failure to recruit or retain participants^3,5^ or lack of generalisability due to being biased towards a certain population (e.g. towards individuals who are more able to attend in-person study visits).^3,6^ Therefore, initiatives are being developed to optimise the efficiency of the conduct of RCTs; decentralised clinical trials (DCTs) being one of these innovations.^7–9^

DCTs are defined as trials in which different elements of the trial such as recruitment, delivery and administration of interventions, study visits, assessment of outcomes and data collection are executed remotely.^10,11^ They obviate the need to travel to a trial centre for participants, and therefore, enable participation from different locations by people who may not have been able to participate in the trial otherwise.^10,11^ DCTs frequently rely on digital and mobile technologies, allowing for more flexible assessments that are not bound by the limitations of scheduled on-site study visits.^10^ A transition from conventional, centralised RCTs to DCTs was on the horizon prior to the COVID-19 pandemic,^7,8,10^ but the demand for such evolution in the design and conduct of RCTs has been recognised more widely during the pandemic and some of their techniques have been rapidly adopted.^12–14^

RCTs play an important role in multiple sclerosis (MS) research as new disease-modifying therapies (DMTs) and symptomatic treatments are still required. In this paper, we review the existing evidence and gaps in knowledge in designing and conducting DCTs in MS research.

After the parameters and scope of the review were agreed by the authors, PubMed and Google Scholar databases and the Google search engine were searched through July 2021 using the keywords (in different combinations) ‘decentralised (or decentralized), randomised (or randomized) controlled trial (or clinical trial or trial), remote, digital, virtual, online, and electronic’ and ‘multiple sclerosis’. For each section, outlined in the review, additional keywords, corresponding to each topic, were used for a more targeted search. All relevant articles and the references cited in these articles were reviewed. If MS-specific articles for any of the sections were considered insufficient, a similar search was performed after excluding the keyword ‘multiple sclerosis’ to find relevant articles from other fields of neurology or medicine.

## Conceptual framework

To ensure that RCTs are appropriately powered for testing the efficacy of a treatment within a limited sample size, they are performed under controlled circumstances where participants tend to have homogeneous characteristics.^15^ Therefore, the findings of RCTs are typically not generalisable, and trials of treatments in real-world populations and under usual clinical practice settings are required to test their effectiveness.^15–17^ Trial designs are moving towards integrating efficacy and effectiveness studies to save time and cost.^15^ DCTs can help reduce this efficacy–effectiveness gap by enabling the conduct of pragmatic trials on a larger number of participants with more heterogeneous demographic and clinical characteristics from different locations and practice settings.^14,15^

RCTs also examine the efficiency of therapeutic interventions, that is, their cost-effectiveness.^18^ There are benefits to undertaking such economic analysis as part of RCTs, such as using prospectively collected patient-level data rather than performing retrospective population studies, but there are also limitations,^18,19^ which could be overcome through DCTs. Conventional RCTs may fail to take real-world costs of a treatment into account.^18,20^ Since extensions of RCTs can be expensive and demanding for both investigators and participants, the follow-up duration of most conventional RCTs are often too short to collect patient-level data on long-term indirect costs of treatment,^18^ such as costs of monitoring MS DMTs, switching MS DMTs or disruptions in their use, their side effects, disability progression due to MS, lost productivity, relapses and hospitalisations.^20^ Also, the cost-effectiveness of an MS DMT estimated in a centralised RCT of a few centres may not be applicable to other healthcare settings due to their lack of generalisability.^18^ Although DCTs cannot eliminate all these problems, they can improve estimations of cost-effectiveness by enabling incorporation of real-world data into RCTs, allowing for long-term follow-up, and increasing the generalisability of their findings.^21^ The costs and savings of applying remote and digital techniques in administration and monitoring of interventions should be carefully calculated when assessing the cost-effectiveness of a proposed treatment in a DCT.

## Recruitment, retention and study population

MS already imposes a high burden on patients by adversely affecting their health and productivity and demanding that a substantial proportion of their time is dedicated to their clinical care.^22,23^ Participating in trials can further disrupt participants’ daily routine and they may incur indirect costs, such as arranging a caregiver.^24,25^ Difficulties of transport to the study site or having other commitments appear to be the main reasons for declining participation in, or withdrawal from, a study.^26,27^ Therefore, RCTs commonly recruit participants at a slower rate than planned or lose participants to follow-up.^5,28,29^ Insufficient recruitment and retention can lead to delays in trial completion, additional costs, underpowered and biased results or premature trial termination.^24,28,30,31^ The same issues can also lead to the inadvertent exclusion of some people with disabilities, multiple comorbidities, or caring or job responsibilities, or people who live far away from, often urban, study sites^3,32^ and reduce the generalisability of the findings.^6^

DCTs can improve participation in studies and retention of participants by allowing them to engage in research activities without the need to travel to a study site and to undertake these activities at their convenience based on their personal and daily schedule.^10,32,33^ For example, people who are unable to walk may be excluded from conventional RCTs, and their participation can be facilitated through DCTs. Therefore, DCTs can include a more diverse group of participants, improving the trial’s generalisability and reducing bias.^34^ For example, MS patients managed in community health services and those managed in specialist MS clinics can be different populations. The findings of a conventional RCT, which tends to recruit participants from MS clinics and hospital settings, may not be generalisable to the broader MS population.^35^ DCTs can be leveraged to enrich recruitment by targeting these underrepresented populations in conventional RCTs. Larger study populations may, however, be required because of the heterogeneous study population and increased variability in outcomes,^36,37^ but this may be a reasonable trade-off for improving the external validity of a trial. The growing use of electronic health records will also facilitate confirmation of diagnosis and review of eligibility criteria during recruitment.

There is a risk that people who prefer in-person interactions or are unable to use digital technologies – for example, due to technological illiteracy, physical disabilities, cognitive or visual problems or lack of resources to support the use of such technologies (e.g. high-speed Internet connections), may still be excluded from DCTs.^38,39^ Advancements in technologies may enhance the usability of digital tools for certain populations. In some circumstances, willing friends or family members could be trained to assist participants with completion of their trial activities remotely. Trials may need to consider more complex hybrid designs, which provide both remote and on-site options, to ensure that their study population is representative of the real-world patient population.

MS trials of therapeutic interventions rarely require the identification of participants in inpatient settings. However, RCTs of some acute inpatient treatments, for example, management of severe disabling relapses, will inevitably require recruitment of participants within inpatient settings with remote follow-up, hence, adopting a hybrid approach to RCTs. Moreover, trials that involve imaging outcome measures are more likely to require hybrid designs.

## Study visits

The growing use of telehealth and e-health tools in routine care of people with MS facilitates the shift towards remote study visits in RCTs.^40,41^ For example, these tools are already being used for providing information regarding a study and remote consenting, including real-time interaction between potential participants and the research staff to ensure that an informed decision is made.^42,43^ The digitisation of other components of a study visit will be reviewed in the following sections.

## Outcome measures

### Clinical

The prospect of digitising outcome measures has played a role in envisaging a future where DCTs are practical.^44^ We report on how digital technologies can reshape RCTs but the specifics of each digitised outcome measure are beyond the scope of this review.

Several existing outcome measures are being or have been converted into tele- or digital assessments to enable remote monitoring of participants and providing them with flexibility in timing their research activities (e.g. the Expanded Disability Status Scale or the Multiple Sclerosis Functional Composite).^39,44,45^ This approach allows for more frequent and even continuous assessments (as opposed to infrequent in-person study visits that tend to be restricted by time), leading to increased power of a study.

People with MS commonly experience fluctuations in their physical and cognitive performance, sometimes exacerbated by the fatigue associated with travel to study sites, which can affect the findings of a trial depending on participants’ performance capacity at the time of testing.^38,46^ Repeated measurements can, therefore, be more realistic and closer to participants’ natural performance compared to cross-sectional assessments.^38,46^ Monitoring composite outcomes in real-time allows for a more dynamic analysis that accounts for the potential relationship between different health-related outcomes,^47^ for example, the effects of participants’ fatigue, pain or mood on their mobility. Real-time recording of patient-reported outcomes not only prevents recall bias, which is likely to occur with retrospective reporting during study visits, but also enables the integration of subjective perceptions of symptoms and objective measurements (e.g. detecting fever during a presumed MS relapse).^48^ E-health and telehealth technologies can improve reporting MS relapses or adverse events in a DCT. The ease and frequency of evaluations in a DCT may, however, lead to over-reporting of side effects compared to conventional RCTs.^44^

Furthermore, the emerging digital evolution in the provision of healthcare presents an opportunity to use routinely collected clinical data in DCTs.^44^ Linking electronic health records to electronic records of RCTs will enable the use of real-world data and outcomes, such as hospital admissions and potential adverse events, which might, otherwise, go unreported.^49^

The digital era has also unlocked opportunities to develop new outcome measures or to assess additional aspects of participants’ performance when using existing ones.^38,39^ Portable and wearable devices, such as smartphones and smartwatches, enable measurement of participants’ physical activity through both passive monitoring and active instructed tests,^28,39,48^ and their use appears to be acceptable to people with MS.^28^ These technologies not only capture conventional measures of physical disability in MS, such as mobility or dexterity, but also introduce objective measurements of other aspects of physical health, such as falls, fatigue, sleep and autonomic dysfunction, which commonly affect the quality of life of people with MS but can be invisible or difficult to capture in conventional RCTs.^39,48^ The application of wearable sensors, however, goes beyond the quantification of physical and physiological features and is also being considered for measuring biomarkers in bodily fluids.^50^ Digital tools also allow the assessment of participants’ learning curves during repeated tests (e.g. Trail Making Tests A and B, Ishihara test, n-back task and 9-Hole Peg test) to evaluate their ability to learn a task and their response speed in addition to response accuracy.^38^

Digital tools and their remote application will require standardisation and validation before their introduction into RCTs,^13,51^ which is being addressed by a growing number of MS-specific studies in recent years.^39,48^ Although the outlook for using digital outcome measures is promising, they can still overburden participants with excessive and complex tasks.^32^ Research staff often directly oversee the completion of outcome measures during in-person study visits, which improves compliance. While data collection could be negatively affected due to poor compliance of participants when they are asked to report outcome measures remotely, routine checks for compliance (e.g. automated emails that go out if an outcome measure is not completed, followed by personnel contact at the next level) can be built into the structure of DCTs to prevent it. Research staff may need to spend more time following up on missing or invalid data with remote compared to on-site data collection. So, it remains possible that the convenience of DCTs will be offset by the inconvenience of the process of remote data validation.

### Imaging

Magnetic resonance imaging (MRI) is one of the most widely used tools in RCTs of MS DMTs.^51^ The use of MRI in a trial may limit decentralisation as participants need to travel to a study site to undergo scans. Mobile and community-based MRI scanners are available,^52^ and can improve participants’ access. Developing and implementing standardised MRI protocols across sites, enabling participants to be scanned at the closest centre, is a practical solution.^53^ The use of standardised MRI protocols for MS diagnosis and follow-up is being advanced by international MS associations.^53^ They are developing strategies to overcome its challenges, such as scanner differences or engagement of different MRI centres, which can also be employed in MS research.

## Therapeutic interventions

Currently, most RCTs of therapeutic interventions in MS that are conducted remotely involve rehabilitation or psychotherapy.^39^ To the best of our knowledge, there are no entirely remote RCTs of pharmacological interventions in MS; our search within clinical trial registries (clinicaltrials.gov and the ISRCTN registry) did not reveal any such studies. Although the remote administration and monitoring of rehabilitation or psychotherapy is facilitated through readily available e-health or telehealth technologies, which are currently being used,^39,40,54^ this is not yet applicable to pharmacological interventions such as DMTs. Pharmacies are increasingly providing drug delivery services to patients’ homes,^55^ but the delivery and administration of some investigational medicinal products can be difficult to undertake entirely remotely; they may require specialised handling during delivery (e.g. cold chain management) or close monitoring during administration.^10^

The administration of some treatments, such as drug infusions, must be monitored by healthcare providers, but could be conducted in home settings. Some local healthcare providers already offer these services to people with MS and can be utilised in DCTs involving altered administration of established DMTs (e.g. extended interval dosing of natalizumab).^56^ Home visits are an alternative approach (e.g. cardiac monitoring at fingolimod initiation or home administration of steroids for relapses);^57,58^ however, the application of these methods to improve participants’ access to trials of investigational medicinal products will require the establishment of dedicated local or mobile research centres.

Digital technologies can be employed for remote monitoring of medication usage and measuring adherence. Direct monitoring of participants’ adherence to a medication by the research staff can be laborious and expensive, and reporting of drug usage by participants can be unreliable.^59^ Digital tools, such as electronic needle disposal systems, electronic pill bottles or electronic diaries enable objective and real-time monitoring of medication usage,^39^ which along with electronic drug reminders can improve adherence.^39,59^

## Data protection

It is evident that the General Data Protection Regulation and other data privacy regulations will also apply to DCTs, but additional considerations regarding data safety and security during their collection, transfer, handling, use and storage will be required for these trials.^10,60^ While the specifics of these regulations are beyond the scope of this review, some examples include policies for using passive data, linking multiple sources of data and ensuring data security on mobile technologies as well as during their transfer in the complicated process of data flow in DCTs.^60,61^

Although digital technologies, through strategies discussed above, present an opportunity to reduce missing data in an RCT, clear instructions on data management need to be included in study protocols to avoid data loss.^10,60^

## Ethics

Institutional Review Boards (IRBs) may be unfamiliar with some approaches that are used in DCTs and have not been widely implemented in trials. As a result, the ethical and regulatory review process for a DCT may be prolonged compared to a conventional RCT. Regulatory bodies and researchers need to work closely with IRBs to ensure that DCTs meet all the criteria for ethical research.

## Study sites and setup

It is likely that as centralised RCTs evolve into DCTs, the organisation of study sites will transform as well. Local clinical trial hubs and mobile facilities run by a network of clinical research employees could still perform research activities that cannot currently be done remotely (e.g. MRI scans, sample collections and drug administration). Remote conduct of RCTs can facilitate more widespread involvement of smaller study sites in trials.^13^

Remote study site initiation and staff training has commonly been used during the COVID-19 pandemic, and might be preferred, because it saves time and cost.^62^ It is important to ensure that the research staff are trained appropriately for their roles in a DCT, which will entail different responsibilities compared to a conventional RCT (e.g. management of electronic, instead of manual data entries or training participants to use digital tools).^13^

Digital tools should be made user-friendly and run efficiently so that the research staff are not overburdened by tackling technical problems.^32^ Implementing a technical core or help centre into the structure of DCTs may alleviate the pressure on research staff.

## Costs

RCTs are expensive and digitising them is thought to reduce their cost.^63^ A 2011 study showed that decentralised trials have higher data management costs than centralised trials.^64^ Although reduced in-person study visits in DCTs will save costs, the added costs of the remote approaches discussed above are study specific. It is likely that advancements in digital technologies (e.g. unified rather than local data storage) and their more widespread use will reduce these costs. Also, the reduced risk of delays in trial completion or its failure is probably an economic advantage of DCTs over conventional RCTs. The evidence regarding the costs of DCTs compared to conventional RCTs is limited, however, and may change over time with developments in DCT designs and their widespread application.

## Implementation

The aim of implementation research is to narrow the gap between finding an efficacious and effective intervention and its evidence-based use in clinical practice.^65^ Implementation strategies are increasingly being explored within trials to accelerate this process.^65^ DCTs will involve remote and potentially novel modes of administering and monitoring treatments that might have not been introduced into routine care. DCTs could demonstrate the feasibility of certain remote processes that could be adopted to introduce efficiencies in clinical practice. Considering implementation issues at early stages of a DCT is vital to ensure that the intervention can be delivered in clinical practice and to identify adaptations required to achieve the same level of effectiveness.

## Conclusion

Clinical trial designs continue to evolve with the aim of improving efficiency and robustness. Advancements in digital and mobile technologies in recent years have facilitated this process and initiated what we think is a gradual transformation from centralised to decentralised RCTs. DCTs have the potential to increase the statistical power of RCTs, produce more generalisable and less biased results and run more efficiently compared to conventional RCTs by recruiting large heterogeneous study samples, more frequent assessments of outcome measures, capturing participants’ real-world performance and timely trial completion. Organisations have started projects to develop and improve the design and conduct of DCTs.^7–10^

DCTs, however, may not be applicable in all circumstances and, therefore, hybrid approaches are also likely to be implemented. Full transition to DCTs may not be immediately possible as some methods discussed in this review need further validation before their widespread application in trials. However, these are times of great opportunities to adjust and improve clinical trials to better serve our patients.
